# Duodenocaval Fistula in a Patient with Inferior Vena Cava Leiomyosarcoma Treated by Surgical Resection and Caval Polytetrafluoroethylene Prosthesis

**DOI:** 10.1155/2015/575961

**Published:** 2015-06-21

**Authors:** Davide Ippolito, Giulia Querques, Silvia Girolama Drago, Pietro Andrea Bonaffini, Sandro Sironi

**Affiliations:** ^1^School of Medicine, University of Milano-Bicocca, Via Pergolesi 33, 20900 Monza, Italy; ^2^Department of Diagnostic Radiology, H. S. Gerardo Monza, Via Pergolesi 33, 20900 Monza, Italy

## Abstract

Inferior vena cava (IVC) leiomyosarcoma represents an extremely rare disease that commonly involves the segment between the inflow of the renal veins and the inflow of the hepatic veins (46% of cases). We report the case of a patient affected by an IVC leiomyosarcoma, treated with surgical resection, caval reconstruction with polytetrafluoroethylene (PTFE), and right nephrectomy, followed by external beam radiotherapy. Oncological follow-up was negative for 17 years after this combined treatment, since the patient developed a duodenocaval fistula (DCF).

## 1. Introduction

Inferior vena cava (IVC) leiomyosarcoma represents an extremely rare mesenchymal tumor, with approximately 300 cases reported during the last years [1].

This neoplasm can occur in three different segments of the IVC: segments I (below the inflow of the renal veins, 38%), II (between the inflow of the renal veins and the inflow of the hepatic veins, excluded), which is the most commonly affected location (46%), and III (from the inflow of the hepatic veins up to the right atrium, 16%) [2, 3].

Being a slow-growing malignancy, clinical manifestations of leiomyosarcoma typically have a delayed appearance and are commonly represented by abdominal pain (66%) [4], palpable mass, lower limb edema, weight loss, fever, weakness, and Budd-Chiari syndrome [2].

However, even with extensive caval involvement, severe venous obstructive symptoms are not often present due to the development of extensive venous collaterals, which maintain adequate flow around the level of obstruction [5, 6].

Therapy of IVC leiomyosarcoma consists of standard wide surgical resection of tumor* en bloc* with the affected portion of the IVC; this should attain to the goals of complete excision of the tumor and preservation of venous return. The prosthetic substitution of the involved IVC tract is generally performed using a polytetrafluoroethylene (PTFE) prosthesis [7].

Additional right nephrectomy is frequently required for cases involving IVC segment II, even if the kidney is not directly involved [8].

The prognosis of patients affected by IVC leiomyosarcoma is not invariably poor, with 3- and 5-year disease-specific survival rates of approximately 75 and 35%, respectively. Up to 50% of patients show recurrence, typically within 30 months after resection [5].

Duodenocaval fistula (DCF) is a rare [9] but lethal postsurgical complication [10, 11]. Trauma is the most common etiology (50%) but DCF might also arise following migration of IVC filters (27%), transmural migration of ingested foreign bodies (16%), or peptic ulcer disease related to retroperitoneal tumor resection in association with radiotherapy (19%) [9].

Diagnosis of DCF is challenging and rarely obtained before laparotomy or autopsy (50%) [4], because symptoms are nonspecific. The most common clinical presentations are sepsis or gastrointestinal hemorrhage (70%) [9]. The high mortality rate (40%) has been attributed to the difficulty of diagnosis, therefore reducing attempts to definitive treatment [9]. CT provides a noninvasive evaluation of the IVC and the adjacent structures, allowing a correct identification of DCF in approximately 50% of patients [12]. CT is able to detect thrombus and air bubbles in the IVC and infectious fluid collections or abscess around the IVC and the duodenum, also allowing the evaluation of collateral vascular outflow [12, 13]. Using conventional or flow-sensitive sequences, MRI can clearly demonstrate high-signal enteric contrast medium or thrombus and signal-void air bubbles within the IVC [10]. Also cavography can reveal thrombus or filling defects in the IVC (33% of cases). Endoscopy typically discloses a duodenal ulcer that may show visible bleeding in 30% of patients but the extent of penetration could be underestimated [14].

In the current case report, we present a patient with leiomyosarcoma, treated with IVC resection and right nephrectomy, followed by reconstruction with PTFE and external beam radiotherapy, who developed a DCF 17 years later. The patient was admitted to our emergency department for abdominal pain and small bowel obstruction, but no signs of sepsis or massive bleeding were found because the prosthesis collapsed.

We aimed at reporting our experience in the management of DCF, describing clinical presentation, possible etiologies, and therapeutic implications, and focusing on the diagnostic challenges in our case.

## 2. Case Report

Our institutional review board approved the preparation of this case report. In August 2013, an 82-year-old woman presented to our emergency department after 1-week persistent vomiting, with a concomitant diagnosis of duodenitis and gastrectasia by esophagogastroduodenoscopy (EGDS) and contrast radiograms performed in another hospital which did not report any signs of ulcer. Moreover, no signs or symptoms of infection were reported by the clinicians.

Her past medical history was positive for colic diverticulosis, cholecystectomy, bilateral hysteroannessiectomy, and excision of a retroperitoneal leiomyosarcoma of the IVC performed in 1996 along with right nephrectomy, IVC reconstruction followed by radiotherapy. Oncological follow-up was negative. She reported multiple episodes of deep venous thrombosis of the right limb due to chronic venous obstruction, treated with ticlopidine (250 mg/day). Additionally, she underwent ileal resection extended for 38 cm from the ileocecal valve, not involving the duodenum, and ileoileal anastomosis for bowel obstruction due to postsurgical adhesive bands in December 2012; however, a late effect of external beam radiotherapy could not be excluded.

**Figure 1:**
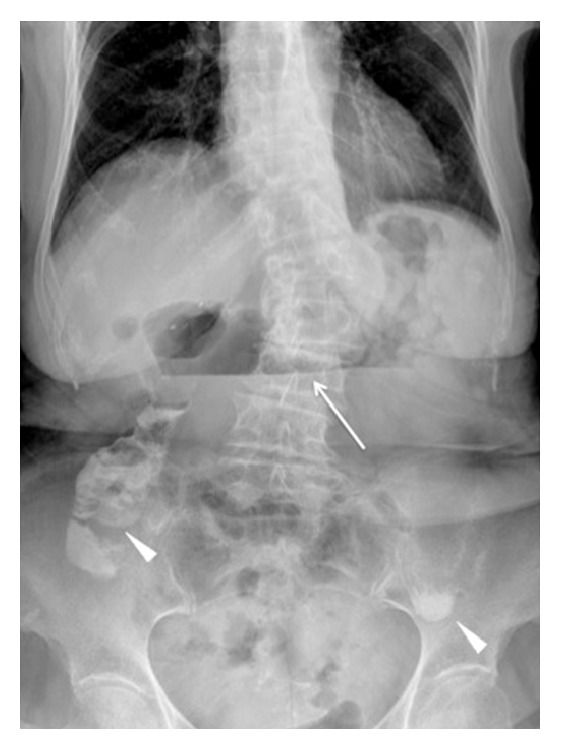
Plain abdominal radiography, performed at the time of emergency admission, shows the presence of a gastroduodenal air-fluid level (white arrow). The partial opacification of residual intestinal lumen (arrowheads) is due to assumption of oral contrast agent in a previous radiographic study performed one week before in another institution.

**Figure 2:**
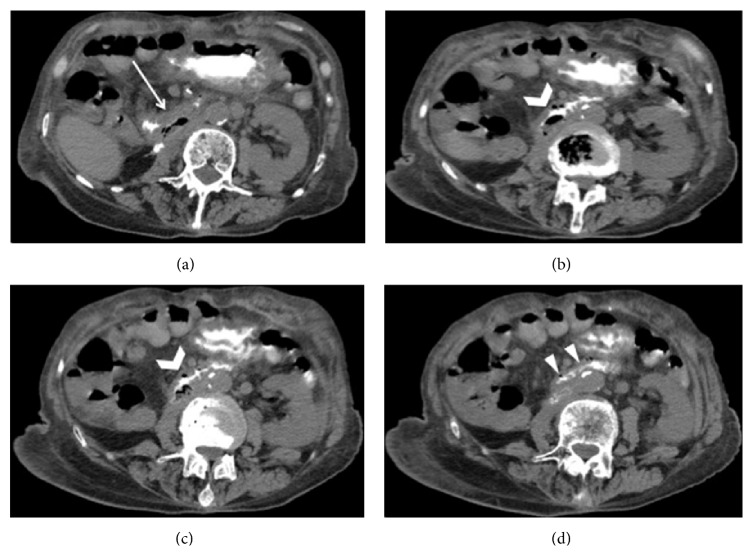
Transverse abdominal unenhanced CT scan acquired after oral administration of a water-soluble contrast agent. The images show the collapse of the second part of the duodenum (white arrow (a)) and the presence of an air bubble collection (open arrow (b) and (c)) (35 × 35 × 15 mm), located between the posterior wall of the duodenum and the collapsed IVC graft. The transaxial CT images of the lower tract of the duodenum demonstrate the trouble of assessing attenuation differences between IVC graft and luminal contrast agent (arrowheads (d)).

At our emergency department plain radiography was initially performed, revealing dilation of the first part of the duodenum and the presence of gastroduodenal air-fluid levels (Figure 1). Hence, the patient was admitted to the surgical department. Then, an unenhanced CT scan was performed on a 16-slice multidetector CT scanner (Brilliance, Philips Medical Systems, Eindhoven, Netherlands), after the oral assumption of a water solution of enteric contrast medium (0.5–1.5 of 3% Gastrografin, Bayer, Milan), in order to rule out the cause of bowel obstruction. The CT images revealed the presence of air bubbles, located between the posterior duodenal wall and the prosthetic IVC, that were interpreted as physiologic air within the intestinal lumen (Figure 2). During the whole hospitalization, the patient remained asymptomatic and was treated with conservative therapy. A semisolid diet was gradually reintroduced and was well tolerated. The patient was then dismissed on August 24, 2013.

**Figure 3:**
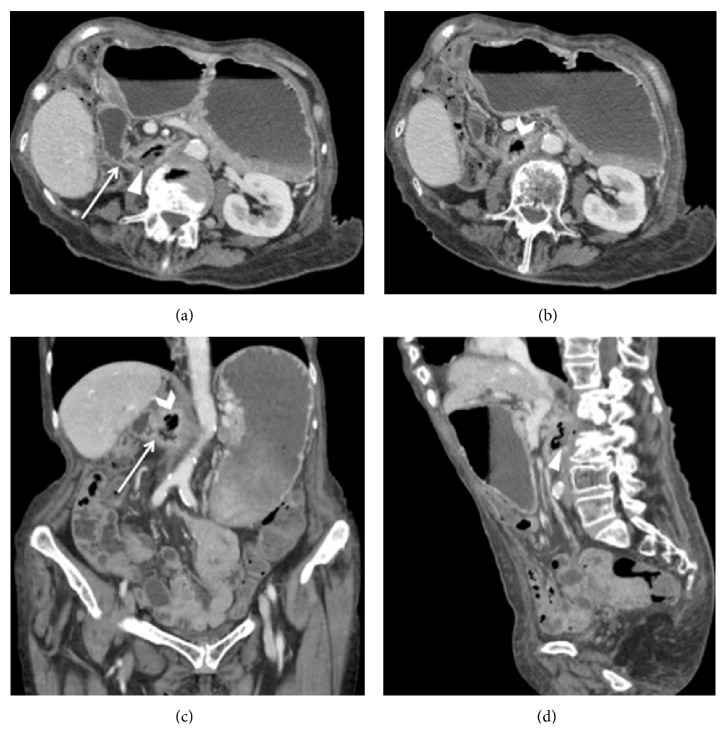
Follow-up contrast-enhanced MDCT study performed three months later. Axial ((a) and (b)) and coronal (c) CT images well show the presence of a fluid-filled fistula (white arrows (a) and (c)) between the duodenum and the IVC graft and an air bubble collection inside the prosthesis (open arrows (b) and (c)). Axial (a) and sagittal (d) CT images better show the path of enteric fluid into the IVC graft. Dilatation of the stomach with an air-fluid level and thickening of the duodenum are also evident.

Three months later, a surveillance follow-up CT study was performed on a 256-slice multidetector scanner (iCT, Philips Medical Systems, Eindhoven, Netherlands). Images were acquired before and after (portal venous phase) the intravenous injection of 100 mL of nonionic iodinated contrast material (Xenetix 350; Guerbet, Aulnay, France) at a flow rate of 3.5 mL/s, using an 18-gauge catheter positioned into an antecubital vein. This study clearly showed a fistula connecting the second part of the duodenum and the prosthetic IVC, along with an incomplete small bowel obstruction and a dilated stomach (Figure 3). Moreover, CT demonstrated a chronic obstruction of the IVC with large collateral veins stemmed from the hemiazygos system and associated with marked dilation of the left ovarian vein. Despite the IVC being obstructed, the patient was asymptomatic and not at risk for bleeding. Thus, no further emergency surgical intervention was required.

## 3. Discussion

Several aspects of our observations are noteworthy. First of all, DCF represents a relatively rare clinical manifestation and only 37 cases have been described [9]. The occurrence of DCF has been reported in different oncological settings, such as retroperitoneal metastatic cholangiocarcinoma [11], renal cell carcinoma [10], urothelial tumor [9], and uterine adenocarcinoma [13]. However, to the best of the authors' knowledge, this represents the first case of a DCF in a patient previously treated for an IVC leiomyosarcoma.

Different factors could have contributed to the development of DCF in this case.

The frequent association of duodenal ulcer with these fistulae suggests that the fistula itself may be related to postirradiation fibrosis and mucosal damage. The adherence of duodenum and IVC portions within an irradiated field, coupled with mucosal damage and fibrosis due to irradiation, might lead to ulceration and fistula formation [9]. As described in literature, radiotherapy has been shown to predispose to the development of DCF several years (on average 26 months, range 6–120 months) after treatment [12, 14]. This could explain the late appearance of this manifestation observed in our patient, who underwent radiotherapy 17 years before.

DCF represents a life-threatening condition, associated with a 40% mortality rate in 37 patients, as reported in a previous review [9]. Because symptoms are nonspecific, diagnosis is mainly based on the results of radiologic and endoscopic studies [9]. CT allows a noninvasive evaluation of the IVC and the adjacent structures [12] and is capable of detecting thrombus, air bubbles in the IVC, or infectious fluid collection around the IVC itself and duodenum [11, 12]. Endoscopy generally demonstrates ulcer of the duodenum, along with visible bleeding, but cannot properly rule out the effective extent of penetration [10, 14]. In our case, duodenitis was diagnosed by endoscopy. However, the thickening of the duodenum wall, the incomplete obstruction of the small bowel, the dilatation of the stomach, and the presence of DCF were identified by CT. Thus, the most important CT finding was a direct tract of gas traced from the involved duodenum towards the IVC graft filled with low-density fluid of intraluminal contents collection (Figure 3).

Finally, it should be highlighted that the occurrence of DCF represents a lethal event and prompt laparotomy is mandatory [11, 15]. Since the IVC was chronically obstructed, no gastrointestinal hemorrhage occurred. Furthermore, there were no signs of deep venous thrombosis, because of the intravenous outflow replacement through the ovarian and hemiazygos veins to the superior vena cava. Thus, no further emergency surgical intervention was required in this patient. Conversely, other literature's series showed that palliative endovascular stent graft [13], prosthesis ablation [11], digital control of bleeding, suturing of the duodenum and IVC, and the placement of an epiploic or jejunal patch to prevent recurrence [16] were necessary.

## 4. Conclusions

Our investigation reports the first case of a DCF related to a previously treated leiomyosarcoma of the IVC. This complication was properly highlighted by contrast-enhanced MDCT, which represents a conventional imaging technique extensively applied in clinical practice. Moreover, although DCF usually represents a lethal event, in our case it had a protracted and indolent natural history due to the chronic obstruction of IVC.
